# Cross-sectoral Food Systems Policy Action for Nutrition: Lessons From National, Regional, and Global Experience

**DOI:** 10.34172/ijhpm.9052

**Published:** 2025-09-28

**Authors:** Anne Marie Thow, Dori Patay, Phillip Baker, Penny Farrell, Erica Reeve

**Affiliations:** ^1^Leeder Centre for Health Policy, Economics and Data, Sydney School of Public Health, The University of Sydney, Sydney, NSW, Australia.; ^2^Sydney School of Public Health, Faculty of Medicine and Health, The University of Sydney, Sydney, NSW, Australia.; ^3^Global Centre for Preventive Health and Nutrition (GLOBE), Institute for Health Transformation, Deakin University, Geelong, VIC, Australia.

**Keywords:** Nutrition, Policy Coherence, Food Systems, Cross-sectoral

## Abstract

**Background:**

Improving nutrition is a global priority for food systems transformation. The introduction of policy measures across multiple sectors relevant to food systems is critical to this transformation. However, integrating measures to improve nutrition into food system policies across multiple government sectors has proved challenging.

**Methods:**

A theory-informed qualitative policy analysis was conducted to identify enablers and barriers of "cross-sectoral" policy action for nutrition in government sectors related to the food system. The analysis drew on interview data (n=43) with policy-makers at global, regional, and national level, in diverse policy sectors, who had experience of engaging successfully across food system policy sectors to improve nutrition.

**Results:**

Success in cross-sectoral policy related to the achievement of nutrition objectives in a way that also enabled achievement of other sectoral objectives, and involved strategic and constructive policy engagement across sectors. Challenges included the need to overcome diverse sectoral mandates and norms, siloed structures of governance, and fluctuations in political interest to engage effectively across sectors for policy change. Key enablers of cross-sectoral policy for nutrition included: supportive institutional structures, such as platforms for engagement, mandates and incentives; ideas that facilitated constructive engagement between policy sectors, including a shared vision, a long-term approach and effective framing; discursive approaches to engagement that balanced multiple interests across policy sectors; and ongoing learning.

**Conclusion:**

This analysis provides new insights to strengthen policy engagement and design more effective capacity building for nutrition policy-makers. This includes "soft skills" that enable effective engagement across sectors and strategic approaches to managing diverse interests influencing policy.

## Background

**Key Messages**

**Implications for policy makers**

- There are fundamental challenges in food systems policy including overcoming diverse sectoral mandates and norms, siloed structures of governance, and fluctuations in political interest.
- This study highlighted important enablers of cross-sectoral policy for nutrition including supportive institutions, shared visions to facilitate constructive cross-sectoral engagement, long-term approaches, effective framing, and ongoing learning.
- There is an opportunity to strengthen institutional mechanisms for engagement, develop shared visions across sectors and learn across sectors and jurisdictions to strengthen policy.

**Implications for the public**

There is an urgent and growing need to make human diets more nutritious, safe and sustainable. This requires that governments integrate nutrition into the considerations of other food system policies, let it be agriculture, economy, trade, or environment. However, this has been challenging to achieve because nutrition is often perceived as a health sector policy issue alone, and there are limited arrangements to facilitate cross-sectoral engagement within governments. This study identified the enablers of ‘cross-sectoral’ policy action to improve nutrition in government sectors related to the food system, by drawing lessons from experiences of success. The enablers highlighted include supportive institutions, shared visions to facilitate constructive cross-sectoral engagement, long-term approaches, effective framing, and ongoing learning. The lessons drawn in this study may inform policy-makers’ efforts worldwide to successfully develop and implement cross-sectoral nutrition policies, which will help ensure access to nutritious, safe, and sustainable diets for all.

At the United Nations (UN) Food Systems Summit in 2021, Heads of State from around the world committed to improving outcomes from food systems.^1^ Nutrition is one key outcome of food systems that has been a long-term global priority, with recent commitments to scale up action under the Sustainable Development Goals and the UN Decade of Action on Nutrition (2016-2025).^2,3^ However, the number of people facing hunger globally rose from 581 million in 2019 to an estimated 733 million in 2023, and 2.8 billion people could not afford a healthy diet in 2022 as a result of rising food prices.^4^ During the same period, severe food insecurity increased globally and in every region.^4^ Efforts towards food systems transformation to improve nutrition and health have been occurring in a rapidly changing context. Supply chain and economic disruptions associated with the COVID-19 pandemic and regional conflicts, for example, have exacerbated negative impacts on food systems already impacted by energy and climate crises.^4^

 Recent calls for transformative changes throughout food systems, to make human diets more nutritious, safe, and sustainable^5,6^ have increased recognition of the need for policy measures that span multiple sectors related to both food systems and health, such as agriculture, environment, and trade.^7,8^ Progress has been made by many governments in adopting best-practice recommendations that improve food systems for nutrition, including through labelling, social protection measures, agriculture policy interventions, school food policies, restrictions on marketing to children, and fiscal policy.^9^ Where progress has been achieved, key contributors have been sustained political commitment for nutrition, the mobilization of civil society groups, the creation of enabling governance bodies and policy frameworks, resourcing and monitoring.^10-12^

 However, progress globally on integrating nutrition into policies across food system sectors remains patchy, and there have been common challenges to translating evidence into policy action across relevant sectors.^13,14^ A particular challenge has been engaging non-health sectors of government in the design and implementation of policy for nutrition, because it is often seen as a health sector policy issue alone.^15^ Priority for nutrition in food systems has been undermined by conflicts of interest, power imbalances, and inequalities in whose voice is heard.^1,16-18^ In addition, commercial food industry actors often strongly oppose nutrition policy measures where they may impact on profitability.^19-21^ Policy-makers also face challenges to policy coherence for nutrition in the form of limited governance mechanisms, low capacities and an absence of mandates and empowerment to support cross-sectoral engagement.^15^

 There is an urgent need to overcome these challenges, and support integration of nutrition into food system policy sectors. The aim of this qualitative policy analysis was to learn from experiences of success in the adoption of policy measures to improve nutrition in government sectors related to the food system, to identify enablers of “cross-sectoral” policy action. Policy sectors relevant to food systems was defined with reference to the Voluntary Guidelines on Food Systems and Nutrition, which outlines agreed policy actions across multiple sectors.^22^ Our definition of policy “success” focussed on incremental improvements to policy to integrate nutrition considerations, with an emphasis on the policy-making process, including political, programmatic, and procedural dimensions, to identify lessons for policy-makers working in the space.^23^ Learning from experience of other jurisdictions can be a valuable source of information for policy-makers, as it can provide insights into policy engagement approaches, strategies to overcome challenges and institutional contexts, that have contributed to successful policy outcomes.^24^ We drew on political economic perspectives on policy-making to examine ideational, institutional, agential, and interest-related enablers that were identified as contributing to cross-sectoral policy success.

## Methods

 The study drew on established methods for policy analysis and case study research,^25^ within a critical-realist paradigm that interprets phenomena as the result of layered realities that need to be critically examined.^26^ The research design drew on theories of the policy process that focus on policy learning and political economy.^27,28^ Based on these theoretical perspectives, key dimensions considered in the design of the interview guide and coding framework included stakeholder interests and influence; political context; the capacities of policy actors; ideas related to policy problems and solutions; and institutional structures (Table 1). As part of the interview, participants were invited to share a specific example of a successful policy initiative for nutrition, that included cooperation and engagement by two or more policy sectors, and examples of potentially relevant sectors were provided, based on the Voluntary Guidelines on Food Systems and Nutrition. The interview guide was piloted with a high-level policy officer with expertise spanning trade, agriculture and food systems.

**Table 1:** Coding Framework

**Interview Guide Themes**	**Codes**	**Sub-codes**
**Barriers or challenges** to cross sectoral policy design, coordination and implementation	Nutrition policy perceptions	
	Challenges	Paradigm/idea challenges
		Institutional challenges
		Evidence challenges
		Context challenges, including political, and other dimensions
	Trade-offs	
**Key approaches** that help to navigate and manage these barriers and trade-offs to improve policy outcomes for nutrition **&The ideal process** through which global policy guidance could support cross sectoral policy action to improve nutrition at national level	Success definition	
	Role of global agencies	Guidance
		Support
		Coordination
		Problems
	Characteristics of actors	Skills and characteristics
		Capacities
**Learnings** from a specific policy initiative, involving more than one sector working together for improved nutrition	Learning	Process lessons
		Institution and mandate lessons
		Resource lessons
		Evidence lessons
		Political lessons
		Policy design lessons
		Communication lessons
		Other lessons

 Between May and September 2023, the lead author conducted 43 semi-structured interviews with public policy actors who work at global, regional or national level in mid-career or senior positions (response rate 65%). Interviewees were identified through a combination of purposive sampling based on (1) knowledge and experience related to the practice of cross-sectoral nutrition policy with a focus on food systems sectors; and (2) understanding of transferability of experience and lessons across economies and governance levels, and snowball sampling (interviewees were asked to identify other potentially relevant interviewees), and contacted via email. For one country in each region with experience of success in nutrition policy across sectors, a second interviewee was sought, to increase robustness of the data collected. The research was partly conducted in conjunction with a Fellowship position at the Committee on World Food Security, which enabled co-design and familiarity with the study context. The sampling was aided by the “insider” nature of the lead researcher^29^; 25 of the interviewees were known professionally by the researcher. The lead researcher was careful to adhere to ethical principles in conducting the research, including consent, confidentiality, voluntariness, and transparency. The interviews were conducted in person (n = 11) and via an online platform (n = 32), lasted for one hour (range 50-75 minutes) and were recorded and transcribed in full.

 The interview data were analysed iteratively. First, all the notes made during interviews were reviewed by the lead author, and a detailed summary was written up with key themes identified. A codebook was prepared with deductive and inductive codes (Table 2), and the data were coded in NVIVO^TM^. The lead author conducted a first round of thematic inductive analysis of the coded data, which focussed on understanding findings across codes related to the definition of policy success and factors enabling cross-sectoral policy. A summary of preliminary findings was sent to all interviewees with a request for feedback, to both inform policy-making moving forward and to inform further focussed analysis of data. The feedback received from 7 interviewees was then integrated into the subsequent phase of analysis. The lead author contacted the co-authors to contribute to a second phase of analysis of these coded data, due to their expertise in nutrition policy and governance. The authorship team met for 3 hybrid analysis workshops, at which they discussed the ways the factors identified interact, shape and are dependent on each other and identified relevant concepts from political economy approaches to policy analysis,^27,28^ which emphasise the influence of institutions, ideas and interests on policy-making as well as the dynamic interaction between policy actors and institutional structures and functions. The workshops were complemented by written contributions to the analysis from all co-authors, which were discussed and refined at subsequent workshops. The Results presented here reflect the findings of this secondary analysis, structured to summarise key factors contributing to success.

**Table 2:** Key Characteristics of Interviewees

**Jurisdictional Level(n = 43)**	**Primary Expertise (n = 43)**	**Location(n = 43)**	**Summary of the Range of Case Study Policies Detailedby Interviewees**
Multilateral (n = 25), Regional (n = 5), National (n = 13)	Nutrition policy (n = 23), Macro-economic policy (namely trade and finance) (n = 5), Agriculture policy (n = 4), Environment and/or social policy (n = 4), Explicit expertise and mandate in food systems policy (n = 3), Foreign policy (n = 4)	Multilateral agencies (n = 25), Latin America (2 at regional level, 2 in Chile, 1 in Ecuador), Africa (1 at regional level, 2 in Ghana, 1 in Zimbabwe), Europe (1 regional, 2 in the UK and 1 in Germany), Asia-Pacific (1 at regional level, 2 in Thailand, 1 in Nepal and 1 in South Korea)	*Jurisdictions:* Barbados, Bhutan, Brazil, Chile, Ecuador, Egypt, Germany, Ghana, India, Korea, Lesotho, Mexico, Mongolia, Nigeria, Portugal, Sierra Leone, Somalia, South Africa, Spain, Thailand, United Kingdom, Zambia, and Zimbabwe *Policy areas:* School meals, child poverty and malnutrition, front-of-pack nutrition labelling, food and nutrition composition tables, marketing restrictions, multisectoral nutrition action plan or policy, coordination structure, city level food governance, climate action and nutrition, nutrition-related tax, trans-fatty acid removal, agriculture and nutrition, fortification, social welfare and nutrition, sodium reduction

## Results

 Altogether, 43 participants were interviewed across different jurisdictional levels, expertise, and geographic location (Table 2). Around two-thirds of the interviewees had experience with a primary mandate to work towards or lead on developing nutrition policy (in Health or Agriculture), including engagement with “other” sectors. Around one-third were situated within the “other” sector, with nutrition-relevant experience but a different primary policy mandate (education, livelihoods, environmental sustainability, economic growth, agricultural and fisheries production, trade and industry development, and diplomatic objectives).

###  Defining “Success” in Cross-sectoral Policy, and Understanding Challenges to Achieving Success

 Interviewees articulated success in cross-sectoral policy as being able to achieve nutrition objectives, in a way that enabled (or at least permitted) the achievement of objectives of the “other” sector(s), which usually did not have a strong nutrition mandate. In their reflections on policy “success,” interviewees described an interplay between agency and structure in which strategic action by policy-makers, supported by institutional structures, resulted in constructive policy engagement between sectors and the design and implementation of cross-sectoral policies for nutrition. The focus of the results presented here is on enablers of cross-sectoral engagement and policy change, as pathways through which power (implicitly) was exerted, with reference to key theoretical constructs of institutions, ideas and interests.

 The most common challenges to cross-sectoral engagement cited by interviewees also reflected an interplay between institutions, ideas and interests. Institutional challenges included the rigidity in (siloed) institutional structures and historical contexts, which located policy responsibility for nutrition within the health sector (only). These were closely linked to the challenges around ideas and paradigms related to the perceived complexity of nutrition and the different ways of working and paradigms across policy sectors. Challenges around interests were based on the perception of limited evidence for co-benefits as well as long causal pathways from food systems change to nutrition outcomes, tensions between economic and nutrition objectives in relation to food systems, fluctuations in political interest in nutrition, a lack of incentives for cross-sectoral policy engagement and coordination and competing priorities for time and resources. The interviewees also identified enablers to successful cross-sectoral nutrition policy-making, described below and summarised in Table 3 with reference to key theoretical constructs of institutions, ideas and interests.

**Table 3:** Enablers of Cross-sectoral Nutrition Policy Success

**Institutional Enablers**	Interviewee References*
Supportive institutional structures for multisectoral coordination	#1, #10, #11, #13, #21, #22, #24
Multisectoral coordination platforms on nutrition	#1, #3, #17, #20, #22, #24, #25, #32, #35, #39, #40, #43
Locating the platform outside of sectoral domains	#17, #20, #24, #39, #43
Coordination platforms at sub-national level	#4, #17, #21, #26
Informal discussion and engagement via ad-hoc, solutions-focussed meetings	#6, #13, #17, #19, #31, #32, #37, #41
Mandate from a higher administrative or political level	#1, #9, #10, #11, #13, #14, #15, #17, #18, #22, #24, #29, #34, #38
Embedding this mandate into institutional incentives	#15, #18, #17, #19, #25, #27, #29, #38
Embedding this mandate into structural incentives related to budgeting, reporting and accountability	#10, #17, #19, #23, #36
Shared budgets and budgetary coordination	#3, #4, #9, #12, #17, #19, #22, #24, #29, #34, #35, #36, #37, #41, #43
Accountability mechanisms	#3, #7, #12, #17, #19, #20, #24, #29, #35, #36, #40, #41
Clarifying sectoral roles and responsibilities	#11, #15, #22, #24, #27, #29, #31, #37, #9, #39, #34
**Ideational Enablers**	
Shared vision for benefits	#2, #7, #9, #17, #18, #23, #27, #30, #40, #41
Conceptualising policy success in the long-term	#1, #2, #10, #11, #15, #16, #31
Compromise is seen as part of the process, not a failure	#2, #10, #16, #37, #38, #9, #31, #11
Effective framing and presentation of evidence, with reference to the relevant sector	#1, #2, #3, #4, #5, #8, #9, #10, #11, #12, #13, #14, #15, #16, #17, #18, #19, #22, #24, #27, #29, #30, #31, #32, #33, #34, #35, #36, #38, #41, #42, #43
Considering nutrition action within a systems framework	#2, #12, #18, #19, #20, #21, #23, #35
**Interest-Related Enablers**	
Spaces for meaningful discourse	#1, #6, #10, #11, #13, #14, #15, #17,#19, #29, #30, #31, #32, #33, #34,#35, #36, #37, #38, #39, #41, #42
Strategic preparation that enabled a balancing of interests	#1, #2, #4, #6, #8, #9, #10, #11, #13, #15, #19, #22, #25, #29, #31, #33, #34, #35, #36, #37, #38, #41, #42
Mediation by senior or political actors	#1, #9, #10, #11, #13, #14, #29, #38, #41
Processes to manage conflicts of interest	#1, #2, #11, #15, #21, #32, #37
**Enablers Arising From the Convergence of Institutional, Ideational, and Interest-Related Drivers**	
Ongoing learning	#2, #4, #10, #11, #15, #16, #18, #19, #25, #27 #33, #34
Shared learning	#2, #18, #19, #27, #31, #33, #34

* Full participant codes indicating sector and geographic scope: #1_nutrition_Thailand; #2_nutrition_global; #3_foreign_policy_global; #4_nutrition_global; #5_trade_global; #6_trade_global; #7_nutrition_global; #8_nutrition_global; #9_nutrition_Chile; #10_nutrition_Chile; #11_nutrition_UK; #12_nutrition_global; #13_trade_global; #14_development_global; #15_agriculture_Germany; #16_nutrition_Korea; #17_nutrition&agriculture_global; #18_foodsystems_global; #19_nutrition_global; #20_foreign_policy_global; #21_environment_global; #22_agriculture_LatinAmerica; #23_nutrition_global; #24_nutrition_Zimbabwe; #25_development_global; #26_development_global; #27_nutrition_Asia; #28_strategic_global; #29_agriculture_global; #30_nutrition_Ecuador; #31_nutrition_Europe; #32_nutrition_Ghana; #33_finance_global; #34_finance_global; #35_foreign_policy_global; #36_nutrition_global; #37_nutrition_Ghana; #38_nutrition_UK; #39_agriculture_Thailand; #40_nutrition_LatinAmerica; #41_development_global; #42_nutrition_Africa; #43_nutrition_Nepal.

###  Supportive Institutions

 Institutional support for cross-sectoral policy had two main components: Institutional structures, and political mandates embedded into institutional incentives. Here, institutions refer to governance arrangements, enabling laws and policy frameworks, and norms of the organisations (governments and multilateral organizations) that the participants work within.

 Institutional structures at national and subnational level can create a platform for successful engagement and coordination of cross-sectoral action. Twelve participants from around the globe cited the importance of multisectoral coordination platforms focussed on nutrition, and interviewees across nutrition, agriculture and foreign policy sectors suggested that locating the platform outside of sectoral domains enabled cross-sectoral engagement, largely because it encouraged shared ownership and removed a situation in which staff of one Ministry were implicitly trying to coordinate other sectors. This could be achieved through co-chairing of the platform by more than one sector or through hosting it at a supra-sectoral level such as the President’s office. For example:

 “*It ended up with the VP’s [Vice President’s] office… which has a coordination function... But then we said we have two co-chairs, Ministry of Health and Ministry of Agriculture…So that none of them is above the other” *(#17_nutrition_agriculture_global).

 To complement the formal coordination structures, informal discussion and engagement via ad-hoc, solutions-focussed meetings were seen as useful. Informal engagement was identified as having synergies with, and enhancing outcomes from, formal mechanisms for coordination by building rapport and helping to resolve points of tension.

 “*So there’s a committee. That I believe helps the regular engagement on a more personal level. … what I have learned works is... to establish a rapport and discuss things beyond committee level so that we see ourselves as doing this together”* (#37_nutrition_national_ghana).

 A mandate from a higher administrative or political level was identified as facilitating collaboration by 14 interviewees in nutrition, trade, agriculture, development, and finance sectors, through creating the necessary convening power to bring together policy and technical staff across different policy portfolios to make decisions and design and implement policy together. Thus, the higher political will, combined with the technical expertise of mid-level policy-makers, helped create a window of opportunity. Doing* “something difficult is easier if you can just blame it on your boss and say, ‘Look, I know nobody wants to work cross-sectorally, but Minister of Agriculture said we have to do it. It’s done.’ It’s just a lot less debate on whether it’s the right thing to do, because your highest level decision maker’s already made that. There’s less debate, and it’s more just operationalizing it” *(#29_agriculture_global).

 “*We had an opportunity, because at that time we had a president who is a medical doctor, and […] She was very convinced about this also, then she encouraged us to go ahead with this law” *(#10_nutrition_national Chile).

 Embedding this mandate into institutional incentives to recognise and support multisectoral activities was critical for it to be operationalised, and had two dimensions. The first was personal incentives related to career development. Eight interviewees from the nutrition, agriculture, food systems, and development sectors highlighted that developing incentives such as recognition through workplans, and key performance indicators for collaboration across sectors, could create personal motivation and mean that cross-sectoral engagement was less a “labour of love.” For example:

 “*Then it’s also incentives about how is this collaborative work rewarded in terms of professional career development? Is this a good stepping stone? … [it’s] important in terms of anchoring it into professionals work plans and giving it the reward and recognition that it would require to become a real incentive” *(#18_foodsystems_global).

 Second was structural incentives related to budgeting, resourcing, reporting and accountability, which were seen by five nutrition policy-makers as supporting collaborations across sectors and helping to overcome the inevitable ebbs and flows in political interest in nutrition. Fifteen participants suggested that shared budgets and budgetary coordination enabled resources for action by all parties involved and entailed processes for prioritisation of actions that would mitigate partners having to compete for resources. For example,

 “*It’s really, really important …how you coordinate the whole budget to impact in food security and nutrition. Because you have a budget in the Ministry of Agriculture, you have a budget for social protection systems, you have a budget for public health systems, you have different budgets. … So sometimes there are some mechanisms in different countries that the budget is management by one institution and this institution or agency has the power to say, ‘Okay, we need to invest in this, in coordination with other agencies in order to have a better impact.’” *(#22_agriculture_regional LatinAmerica).

 In addition, the oversight and engagement of political leaders was seen by 12 interviewees (10 of whom are working globally) as an incentive for action, and important for introducing accountability between sectors. Participants around the world recognised that an important aspect of operationalizing accountability was articulating clear roles for the different sectors, which engendered responsibility as well as giving each sector a clear sense of relevance and competency. For example,

 “*…their role is to bring together the different sectors plans [and]… bring some level of accountability towards meeting the National Food and Nutrition Security goals [as they are]… coordinating the different ministries” *(#24_nutrition_Zimbabwe).

###  Ideas That Enable Constructive Engagement Between Policy Sectors

 The three ways of thinking about underpinning approaches to successful cross-sectoral policy engagement for nutrition were: a shared vision for benefits, conceptualising policy success in the long-term, and effective framing of nutrition with reference to the other sector and within a systems framework.

 First, ten interviewees from the nutrition, food systems and development sectors suggested that a collaboratively developed “vision” across policy sectors created a sense of shared ownership by people working in the different sectors. Around a third of interviewees identified global initiatives that could underpin the process of developing a shared vision to support cross-sectoral engagement on food systems and nutrition: The Sustainable Development Goals and the World Bank’s human capital project. These initiatives were seen as providing a starting point for conceptualising cross-sectoral commonalities and shared interests at the national level. Related to this, two interviewees also identified the importance of broader conceptions of “success” and new metrics to help articulate how action on nutrition could contribute to long-term efforts to shift socio-economic and political systems to be more equitable. For example,

 “*I mean, we would need to transform economic systems. And there are increasingly voices saying …we should be measuring progress differently” *(#23_nutrition_global).

 Second, six nutrition and one agriculture policy-makers explained that conceptualising policy success as a long-term endeavour—fostered patience, and enabled policy-makers to interpret “compromise” in policy design and implementation as creating a way forward, towards success, rather indicating policy failure. For example, changes to policy design that facilitate adoption in the context of diverse interests, such as allowing for staged implementation or “watering down” of the policy settings. These were often explained as strategic compromises, with a view to strengthening policy over the longer term. For example,

 “*It was an eyeopener again to learn that these things really do take time, and people come to the table with different agendas and expectation. It’s the systematic planning and engaging and disagreeing and compromising, if you like, letting go of some of your own interest” *(37_nutrition_national_Ghana).

 Third, the majority of interviewees indicated that effective framing of nutrition included communicating the policy issue as (1) a problem that required cross-sectoral engagement, and (2) an issue that could benefit the “other” sector, using language that resonated with the mandate of the relevant sector. Evidence was identified as critical to substantiate both these framings and in particular, necessary to show the benefits of the nutrition-related policy problem and actions for the other sector(s).

 “*…showing them how much of the burden and thus how much of the benefit sits outside of the health sector…[for example] the economic productivity losses” *(#41_development_global).

 New frames also included thinking about nutrition policy action within a complex systems framework, which was identified by eight interviewees working globally as generating more creative policy solutions for nutrition that could appeal to the objectives of multiple sectors. Moving beyond immediate policy tensions (such as between economic interests and nutrition) by identifying other related issues, (such as environment and gender), and the complex dynamics between multiple sectors could engender additional co-benefits and mitigate trade-offs. For example:

 “*So even if you can’t necessarily find ultimate win-wins purely on an economic trajectory perspective apart from the cost associated with reduced workforce capacity and impacts on health systems, I think there is something about the win-wins around creating that more kind of resilient and environmentally friendly future” *(#19_nutrition_global).

###  Balancing Interests

 Discursive approaches to engagement were identified as enabling policy-makers to balance multiple interests across policy sectors, manage trade-offs and adapt policy responses to address emerging issues. Three key considerations were highlighted: Spaces for discourse to occur, strategic preparation that enabled a balancing of interests, and mediation by senior or political actors.

 Within formal institutional structures as well as more informal contexts, almost all interviewees emphasised the importance of spaces in which dialogue regarding interests, tensions and synergies could occur; meaningful dialogue that resulted in new understanding and policy change. Six interviews stressed the importance of transparency in communicating benefits and trade-offs – ie, not just making a positive case but acknowledging the tensions. For example, ad-hoc, issue-specific discussions, in which different sectors were invited to engage to try to find solutions were identified as complementing formal meetings through addressing issues that were difficult to resolve in the normal order of business. Six interviewees working on nutrition, two on agriculture, and one on development explained that ongoing dialogue enabled cooperative stakeholder engagement on both the technical and political aspects of the policy, and for creating a space for ongoing reflection and learning during policy development and implementation. For example:

 “*It’s good… to have a little bit more of a focus on regular reflection, checking on how things are going…. there undoubtedly needs to be something that kind of forces that sort of conversation, but ideally where there’s support provided to make sure it;s a quality set of discussions and a quality engagement”* (#19_nutrition_global).

 “*If you bring trade and you bring health together to have conversations, then you can try to find solutions to these tensions we are talking about, but if trade remains where they are, health remains where they are, they can work for a hundred years, there’s no way you’re going to have a solution to the tensions. Bringing them together to have frank conversations, sometimes you can find a middle ground” *(#32_nutrition_national_Ghana).

 Strategic preparation for cross-sectoral engagements, mainly by the health sector, was identified by the majority of the interviewees as important to support dialogue across diverse interests, mandates and concerns relevant to food systems and nutrition, and advance nutrition on the relevant policy agendas. This preparation was supported by specific capacities of policy actors (and teams), including capacities to understand different sectoral roles and mandates, communicate understandably (using the right “language”) across sectors, and to manage both administrative and political processes.

 Mediation of the concerns and tensions of different interest groups was highlighted as important by nine participants, particularly through active engagement by high-level or political actorsduring policy processes. A visible presence of high-level or political actors was also seen as contributing to both internal and public support for a specific policy response, which in turn could enable goodwill for a given measure across sectors. For example,

 “*Something that is working very well is having … the president himself engaging in public events … also with public leaders” *(#14_development_global).

 Several interviewees also identified the need for explicit processes and procedures to manage diverse (sometimes competing) interests. The focus of this in the nutrition context was mainly limiting industry engagement to certain parts of the policy process. Three interviewees also explicitly articulated the importance of addressing power imbalances in cross-sectoral governance settings, including clear processes and structures for managing private sector involvement. For example:

 “*We had different round tables or circles. We had an inner circle with the ministries. We had a second circle with the ministries and two of the major associations, one from the consumer side, one from the business side. …We had different circles which we set in place in different steps of the process”* (#15_agriculture_national_Germany).

###  Ongoing Learning

 Interviewees also described how all of these dimensions interact in shaping policy outcomes. In particular, ideas underpinning action were seen as strengthening or undermining institutional support over the long term. In turn, productive discourse across policy sectors was seen as fostering learning which shapes ideas over time. Ongoing learning, ie, the continuous, active process of learning how to collaborate across sectors, was identified by 14 interviewees working in nutrition, agriculture, food systems, development and finance sectors, as a key enabler of success in cross-sectoral policy engagement. This included ongoing negotiation regarding mandates and roles, enabled by institutional support structures, including political mandates and formal “spaces” for engagement. For example:

 “*Defining roles, making them responsible and accountable for specific things I think encourages them and make people feel relevant to the whole process” *(#37_nutrition_national_ghana).

###  Shared Learning

 Five interviewees working globally and two regionally suggested that shared learning, ie, learning together for creative solutions that were effective (“strategic entry points”), was key to enabling policy innovation. For example:

 “*[as we engage with other sectors] we’re asking questions to unpack the conventional wisdom, to some extent. … Is this the best way of using resources? Is this the best outcome for these people and for their children? … We’re really questioning the role of policy” *(#34_finance_global).

 The participants also emphasised the experimental nature of this shared learning process.

 “*So it’s experiential learning in that respect, simple systems thinking and then common co-creation of solutions” *(#25_development_global).

 This shared learning was also facilitated by secondments of staff between different ministries as a mechanism to foster learning that had worked in their context (#16_nutrition_national_Korea).

## Discussion

 Cross-sectoral policy measures that address food system drivers of nutrition are critical for transformative change. The findings of this study highlight the fundamental challenge of overcoming diverse sectoral mandates and norms, siloed structures of governance, and fluctuations in political interest to engage effectively across sectors for policy change. Enabling constructive engagement across sectors is a critical aspect of developing creative policy solutions that balance nutrition as a priority with other aspects of food system policy. Overall, we found that framing nutrition as a long-term project, building soft skills for relationship-forming and influencing (as a means to exert power), creating institutional structures that enabled constructive dialogue, and fostering ongoing learning were key supports for cross-sectoral policy for nutrition.

 This study highlighted the importance of strongly institutionalising multisectoral action on nutrition, and taking a long-term and iterative perspective on success, given how slow-moving policy development and implementation is and how slow-burning nutrition improvements are. This is consistent with previous literature identifying the importance of institutional structures, such as multisectoral coordination platforms, and high-level mandates for cross-sectoral policy.^30,31^ Complementing these more structural dimensions, the enablers of success documented in this study also echo previous findings regarding the importance of leadership by policy actors and the importance of discursive approaches to engagement that enable the balancing of interests and foster creativity and ongoing learning by policy-makers.^12,32^ The findings related to the need for shared vision, strategic communication and persistence by policy-makers resonate with the challenges to multisectoral nutrition policy recently described in the African, South Asian, and Pacific Island regions.^33-37^ In particular, ongoing learning fosters reflexivity, which has been identified as critical for the development of a shared (across sectors) vision for nutrition.^38^ Conceptualising the integration of nutrition policy as innovation across policy sectors can provide a helpful lens to consider the role of policy “change agents” in adapting best practice recommendations into specific policy measures, through effectively communicating the benefits, compatibility and feasibility of nutrition policy measures, with reference to the mandates and norms of food system policy sectors.^39^ Similarly, these findings highlight the global resonance and unique capacities of “boundary-spanning actors” in nutrition policy (albeit with a focus on internal government processes), which has been identified previously in the context of undernutrition policy in the African region.^40^ These findings underscore the need to strengthen capacities in “soft skills” among nutrition policy-makers that are required to confidently engage across sectors and to communicate to political stakeholders.^15^

 This study has provided new insights into the enablers of successful cross-sectoral policy-making for nutrition in a food systems context, informed by insider research across multiple countries and governance levels. This study is limited by the lack of independent validation of the examples of “successful” cross-sectoral policy reported by interviewees and by relying on insights of those within government and multilateral organisations. While this approach enabled insights into the “black box” of policy-making, it is possible that interviewees presented aspirational accounts of policy processes. Further limitations include the small sample size from each jurisdiction and sector, which limited our ability to undertake comparative analysis, as well as the fact that over half the sample consisted of experts in nutrition policy. Although we observed some nuances regarding context and culture in the approach, there was a strong consistency in the themes that emerged.

 This study also sheds light on approaches to manage diverse interests influencing policy, contributing insights to address commercial determinants of health through a policy process lens.^41^ In the food systems and nutrition policy space, one or more of the other sectors involved usually have an economic mandate; for these sectors, the private sector is often one of the important stakeholders to be consulted within policy-making. However, food industry opposition to nutrition policy change has been consistent and coordinated.^42^ Learning from experiences across jurisdictions, and strategies that have supported successful policy action despite resistance, can support and empower policy-makers.^43^

## Acknowledgements

 This study was partly undertaken in conjunction with an unfunded Food and Agriculture Organization (FAO) Fellowship at the UN Committee on World Food Security. The views expressed in this publication are those of the author and do not necessarily reflect the views or policies of the FAO of the UN. The author wishes to thank those who contributed to co-design of the study, and the interviewees for their willingness to share their expertise and to provide feedback on preliminary findings.

## Ethical issues

 The study was granted ethical approval by the University of Sydney Human Research Ethics Committee (#2022/903).

## Conflicts of interest

 Authors declare that they have no conflicts of interest.
